# Receptor for Advanced Glycation End Products Is Involved in LPA_5_-Mediated Brain Damage after a Transient Ischemic Stroke

**DOI:** 10.3390/life11020080

**Published:** 2021-01-22

**Authors:** Arjun Sapkota, Sung Jean Park, Ji Woong Choi

**Affiliations:** College of Pharmacy and Gachon Institute of Pharmaceutical Sciences, Gachon University, Incheon 21936, Korea; sapkotaa07@gmail.com (A.S.); psjnmr@gachon.ac.kr (S.J.P.)

**Keywords:** brain ischemic stroke, LPA_5_, TCLPA5, RAGE, blood–brain barrier, ERK1/2, NF-κB

## Abstract

Lysophosphatidic acid receptor 5 (LPA_5_) has been recently identified as a novel pathogenic factor for brain ischemic stroke. However, its underlying mechanisms remain unclear. Here, we determined whether the receptor for advanced glycation end products (RAGE) could be involved in LPA_5_-mediated brain injuries after ischemic challenge using a mouse model of transient middle cerebral artery occlusion (tMCAO). RAGE was upregulated in the penumbra and ischemic core regions after tMCAO challenge. RAGE upregulation was greater at 3 days than that at 1 day after tMCAO challenge. It was mostly observed in Iba1-immunopositive cells of a post-ischemic brain. Suppressing LPA_5_ activity with its antagonist, TCLPA5, attenuated RAGE upregulation in the penumbra and ischemic core regions, particularly on Iba1-immunopositive cells, of injured brains after tMCAO challenge. It also attenuated blood–brain barrier disruption, one of the core pathogenesis upon RAGE activation, after tMCAO challenge. As an underlying signaling pathways, LPA_5_ could contribute to the activation of ERK1/2 and NF-κB in injured brains after tMCAO challenge. Collectively, the current study suggests that RAGE is a possible mediator for LPA_5_-dependent ischemic brain injury.

## 1. Introduction

Lysophosphatidic acid (LPA) is an important bioactive lipid that can regulate various biological functions by activating its specific six G protein-coupled receptors (LPA_1–6_) [1,2]. LPA receptors are known to play critical roles in the pathogenesis of various diseases [2,3,4]. Thus, several efforts have been made to develop drugs by targeting LPA signaling including its receptors [2,3,4]. In brain ischemic stroke, the importance of receptor-mediated LPA signaling has been suggested [5,6,7,8,9,10]. Amounts of LPA can be increased in both human patients [5] and animals with stroke [6,7]. Moreover, its receptors LPA_1_ [8] and LPA_5_ [9,10] have been demonstrated to contribute to ischemic brain injuries. In particular, LPA_5_ has been suggested to play a pathogenic role in brain ischemic stroke because it can be upregulated in the ischemic core regions after ischemic challenge [9] and suppressing its activity with TCLPA5, a selective LPA_5_ antagonist, can lead to neuroprotection against ischemic brain injuries during both acute and chronic phases [9,10]. However, how LPA_5_ can play its pathogenic role remains unclear.

The receptor for advanced-glycation end products (RAGE) is a multiligand receptor that can bind to advanced glycation end products (AGEs), amyloid beta (Aβ), the S100/calgranulin family, and high mobility group box 1 non-histone DNA-binding protein (HMGB1 or amphoterin) [11]. It has been reported that RAGE plays various pathological roles in regulating cytokines production, Aβ accumulation, protein aggregation, and immune cell infiltration in brain disorders, such as Alzheimer disease, intracerebral hemorrhage (ICH), multiple sclerosis, and brain ischemic stroke [11,12,13]. In particular, in brain ischemic stroke, RAGE can be upregulated in injured brains and either pharmacological or genetic suppression of RAGE activities can attenuate brain injuries [14,15,16]. Such pathogenic roles of RAGE can be associated with proinflammatory cytokine production, NF-κB activation, and gliosis [14,15,16]. In view of the link between RAGE and LPA signaling, it has been previously suggested that RAGE can mediate LPA-dependent biological functions in cancer, such as cancer cell growth in vitro and tumor formation in vivo [17,18]. However, the possible relationship between RAGE and LPA signaling in brain ischemic stroke is unexplored. Moreover, whether LPA receptors could be involved in such a relationship remains unknown.

Here, we addressed whether RAGE could be involved in LPA_5_-mediated pathogenesis of post-ischemic brains with a mouse model of transient middle cerebral artery occlusion (tMCAO). We determined whether LPA_5_ could regulate RAGE expression in injured brains after tMCAO challenge using an LPA_5_ antagonist, TCLPA5. We also determined whether LPA_5_ could contribute to blood–brain barrier (BBB) disruption, one of core pathogenesis regulated by RAGE [15,16,19]. Finally, we determined whether LPA_5_ could contribute to the activation of ERK1/2 and NF-κB, both of which could be effector pathways of RAGE to mediate BBB disruption.

## 2. Materials and Methods

### 2.1. Animals

Male ICR mice were purchased from Orient Bio (Seongnam-Si, Korea). All of the animal experiments were carried out following Institutional Animal Care and Use guidelines of Lee Gil Ya Cancer and Diabetes Institute (LCDI) at Gachon University (approved animal protocol number: LCDI-2019-0027). Mice were housed under a controlled environment with a light cycle of 12 h/12 h of day/night, temperature of 22 ± 2 °C, and a relative humidity of 60 ± 10%. Mice had free access to food and water.

### 2.2. tMCAO Challenge

ICR mice (male, 7 weeks old) were challenged with tMCAO as described previously [9]. Briefly, mice were anesthetized with isoflurane in a mixture of O_2_:N_2_O (1:3) (3% for induction and 1.5% for maintenance). A ventral neck incision was made and the right common carotid artery (CCA) was carefully separated from the vagus nerve. MCAO was induced by inserting a monofilament suture coated with silicon (9 mm long, 5-0) towards the MCA through an internal carotid artery from CCA bifurcation. At 90 min after occlusion, the monofilament was withdrawn to restore the blood flow under an anesthetized condition. For the sham group, mice were subjected to the same surgical procedure except MCAO. Body temperature was maintained at 37 °C during surgery.

### 2.3. Vehicle or TCLPA5 Administration

Mice subjected to the MCAO surgery were randomly divided into a vehicle (10% Cremophor EL and 10% ethanol in distilled water)-administered group or a TCLPA5-administered group. TCLPA5 (5-(3-Chloro-4-cyclohexylphenyl)-1-(3-methoxyphenyl)-1H-pyrazole-3-carboxylic acid, 10 mg/kg, i.p. Tocris Bioscience, Bristol, UK) was administered to mice immediately after reperfusion. TCLPA5 dose was selected based on our previous study [9]. Two mice of the vehicle-administered group were excluded from the study because of death. In the TCLPA5-administered group, three mice were excluded from the study because of death (two mice) or hemorrhage (one mouse). The number of mice per group was 4, 5, and 5 for Western blot, histological, and quantitative real-time PCR (qRT-PCR) analyses, respectively.

### 2.4. Histological Analyses

#### 2.4.1. Brain Tissue Preparation

For histological assessment, brain samples were obtained at 1 day or 3 days after tMCAO challenge. Mice were anesthetized with a mixture of Zoletil 50^®^ (10 mg/kg, i.m., Virbac Laboratories, Carros, France) and Rompun^®^ (3 mg/kg, i.m., Bayer HealthCare LLC, Shawnee Mission, KS, USA), perfused with ice-cold phosphate-buffered saline (PBS), and fixed with 4% paraformaldehyde (PFA) in PBS. Brains were removed, post-fixed in 4% PFA for 24 h, immersed in 30% sucrose solution, frozen in Tissue-Tek Optimal Cutting Temperature compound, and sectioned into 20 µm-thick coronal sections using a cryostat (RD-2230, Roundfin, Liaoning, China).

#### 2.4.2. RAGE Immunohistochemistry

Brain sections were post-fixed with 4% PFA, treated with 0.01 M sodium citrate at 90–100 °C, oxidized with 1% H_2_O_2_, and blocked with 1% fetal bovine serum (FBS) in PBS containing 0.3% Triton X-100. Brain sections were labelled with mouse anti-RAGE (1:200, Santa Cruz Biotechnology, Dallas, TX, USA) overnight at 4 °C followed by an incubation with a biotinylated secondary antibody (1:200, Santa Cruz Biotechnology) for 2 h at room temperature. Tissue sections were then incubated with an ABC reagent (1:100, Vector Laboratories, Burlingame, CA, USA) for 90 min at room temperature. Signals were developed using a 3,3′-diaminobenzidine (DAB) kit (Dako, Santa Clara, CA, USA) for 2 min, washed with water, dehydrated with alcohol and xylene, and mounted with Entellan media (Merck, Darmstadt, Germany).

#### 2.4.3. Detection of Cellular Localization of RAGE by Double Immunofluorescence Staining

Brain sections were labelled with a mouse anti-RAGE primary antibody (1:100, Santa Cruz Biotechnology) along with proper primary antibodies of different cell markers including rabbit anti-Iba1 (1:500, Wako Pure Chemicals, Osaka, Japan), rabbit anti-NeuN (1:200, Millipore), rabbit anti-GFAP (1:500, Abcam), and rat anti-CD31 (1:300, Dianova, Hamburg, Germany) overnight at 4 °C. These labeled sections were then incubated with Cy3- and AF488-conjugated secondary antibodies (1:1000, Jackson ImmunoResearch, West Grove, PA, USA) for 2 h at room temperature, counterstained with 4′,6-diamidino-2-phenylindole (DAPI, Carl Roth, Karlsruhe, Germany), and mounted with VECTASHIELD^®^ (Vector Laboratories).

#### 2.4.4. NF-κB/Iba1 and Claudin-5/CD31 Double Immunofluorescence Staining

For NF-κB and Iba1 double immunofluorescence staining, brain sections were fixed with 4% PFA, rinsed with PBS, and treated with Tris-EDTA solution at 100 °C for 30 min for antigen retrieval. For claudin-5 and CD31 double immunofluorescence staining, sections were treated with 1% NaOH in PBS containing 1% H_2_O_2_ for 20 min for antigen retrieval. Such sections were blocked with 1% FBS containing 0.3% Triton X-100 and incubated with primary antibodies of either mouse anti-NF-κB p65 (1:100, Santa Cruz Biotechnology)/rabbit anti-Iba1(1:500, Wako Pure Chemicals) or rabbit anti-claudin-5 (1:300, Santa Cruz Biotechnology)/rat anti-CD31 (1:300, Dianova, Hamburg, Germany) at 4 °C overnight. Sections were then incubated with AF488- and Cy3-conugated secondary antibodies for 2 h at room temperature, washed with PBS, and mounted with VECTASHIELD^®^.

#### 2.4.5. Image Preparation and Quantification

Brain images were taken from either a bright-field microscope equipped with a DP72 camera (BX53T, Olympus, Japan) or a laser scanning confocal microscope (Eclipse A1 Plus, Nikon, Japan). Representative images were prepared with Adobe Photoshop Elements 8 (Adobe Inc., San Jose, CA, USA). Three different images (600 µm × 600 µm) of each brain region in a mouse were used to quantify numbers of immunopositive cells. Data are provided as mean value of these numbers of immunopositive cells per unit area (mm^2^).

### 2.5. qRT-PCR Analysis

Total RNA was extracted from the ipsilateral brain at 3 days after tMCAO challenge using RNAiso plus (1 mL, Takara, Kusatsu, Japan). For qRT-PCR, 1 µg of total RNA was used to synthesize cDNA using All-in-One First-Strand cDNA Synthesis SuperMix (TransGen Biotech, Haidian, China). qRT-PCR was carried out using a StepOnePlus^TM^ qRT-PCR system (Applied Biosystems, Foster City, CA, USA) with Power SYBR Green PCR master mix (Life Technologies, Carlsbad, CA, USA) and primer sets for β-actin and RAGE. Target mRNA expression levels were then normalized with mouse β-actin and quantified using the 2^−ΔΔCT^ method. Sequences of primers used in this study were as follows: β-actin forward, 5′-AGCCTTCCTTCTTGGGTATG-3′; β-actin reverse, 5′-CTTCTGCATCCTGTCAGCAA-3′; RAGE forward, 5′-ACGAGGATGAGGGCACCTATA-3′; and RAGE reverse, 5′-GTCGTTTTCGCCACAGGATAG-3′.

### 2.6. Western Blot Analysis

Ipsilateral brain hemispheres were removed at 3 days after tMCAO challenge and homogenized in tissue lysis buffer (1 mL, OttimoLyse II, JUBIOTECH, Daejeon, Korea) supplemented with a protease inhibitor cocktail (cOmplete^™^ mini, Roche, Mannheim, Germany). Extracted protein samples (20 µg) were separated on 10% SDS-PAGE, transferred to PVDF membranes, and blocked with 5% skim milk. These membranes were incubated with primary antibodies against RAGE (1:1000, Santa Cruz Biotechnology), total ERK1/2 (1:1000, Cell Signaling Technology, Danvers, MA, USA), phosphorylated ERK1/2 (1:1000, Cell Signaling Technology), and β-actin (1:10,000, Bethyl Laboratories, Montgomery, TX, USA) at 4 °C overnight followed by incubation with respective secondary antibodies (1:10,000, Jackson ImmunoResearch,) at room temperature for 2 h. Protein bands were detected with enhanced chemiluminescence solution (Dongin Biotech Co., Seoul, Korea). Expression levels of targeted protein bands were measured with Image J software (National Institute of Mental Health, Bethesda, MD, USA) and normalized with β-actin.

### 2.7. Statistical Analysis

All statistical analyses were performed using GraphPad Prism 7 (GraphPad Software Inc., La Jolla, CA, USA). Date are presented as mean ± S.E.M. Statistical significance was determined by one-way analysis of variance (ANOVA) followed by a Newman–Keuls post-hoc test for comparisons among groups. Statistical significance was considered when *p* value was less than 0.05.

## 3. Results

### 3.1. RAGE Is Upregulated in the Penumbra and Ischemic Core Regions of Injured Brains, Particularly on Iba1-Positive Cells, after tMCAO Challenge

**Figure 1:**
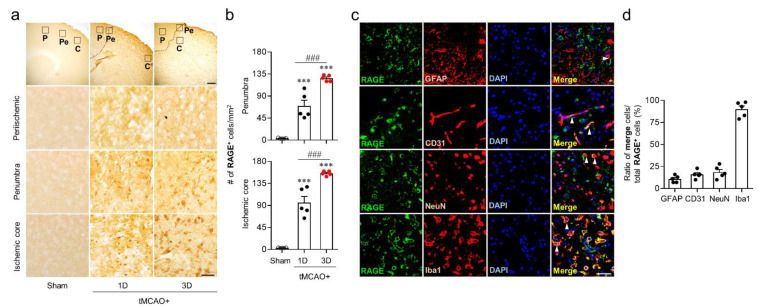
Receptor for advanced glycation end products (RAGE) is upregulated in both penumbra and ischemic core regions, particularly on activated microglia/macrophages, after transient middle cerebral artery occlusion (tMCAO) challenge. Brain samples from sham mice and tMCAO-challenged mice were used to determine expression levels of RAGE in different brain regions and cellular localization of RAGE. (**a**,**b**) RAGE expression levels in the penumbra and ischemic core regions at 1 day (1D) and 3 days (3D) after tMCAO challenge were determined by immunohistochemistry analysis. (**a**) Representative images of RAGE-immunopositive cells in the periischemic (“P”), penumbra (“Pe”), and ischemic core (“C”) regions. Diagram boxes in upper panels display cerebral areas from which middle and bottom panels were obtained. Dotted lines separate periischemic and ischemic core regions. Scale bars, 200 µm for top panel and 50 µm for middle and bottom panels. (**b**) Quantification for the number of RAGE-immunopositive cells in the penumbra and ischemic core regions. (**c**,**d**) Cellular localization of upregulated RAGE in the penumbra (RAGE/GFAP) and the ischemic core regions (RAGE/Iba1; RAGE/NeuN; RAGE/CD31) at 3 days after tMCAO challenge was determined by double immunofluorescence staining analysis. (**c**) Representative images for RAGE expression on activated microglia/macrophages (Iba1), neuron (NeuN), endothelial cell (CD31), or astrocyte (GFAP). (**d**) Quantification of their number in ratio. Arrowheads indicate double immunopositive cells of RAGE and each cell marker. Scale bar, 50 µm. n = 5 mice per group. *** *p* < 0.001 versus sham; ^###^ *p* < 0.001 versus mice at 1 day after tMCAO challenge.

RAGE can be upregulated in human ischemic patients [20] and animals with brain ischemic stroke [15,16,20]. It has been also previously reported that RAGE upregulation can occur in the penumbra region after a permanent brain ischemic stroke [20]. In the current study, we determined region- and time-specific RAGE upregulation by immunohistochemical analysis at 1 day and 3 days after tMCAO challenge. The number of RAGE-positive cells was significantly increased at both 1 day and 3 days after tMCAO challenge compared to that in the sham group (Figure 1a,b). RAGE upregulation was observed in the penumbra and ischemic core regions, but not in the periischemic regions (Figure 1a,b). The degree of RAGE immunoreactivities seemed to be stronger in the ischemic core region than in the penumbra (Figure 1a,b). In addition, the number of RAGE-positive cells was higher at 3 days after tMCAO challenge than that at 1 day after tMCAO challenge (Figure 1a,b). These results indicate that RAGE could be upregulated in lesional areas like the penumbra and ischemic core regions after an ischemic stroke.

Next, we determined in which cell types RAGE upregulation could occur at 3 days after tMCAO challenge by double immunofluorescence staining for RAGE with cell markers Iba1 (activated microglia/macrophage), GFAP (activated astrocyte), NeuN (neuron), and CD31 (endothelial cell). The number of RAGE/Iba1-, RAGE/NeuN-, and RAGE/CD31-double immunopositive cells was analyzed in the ischemic core regions because RAGE upregulation was clearly observed in these regions (Figure 1a,b). The number of RAGE/GFAP-double immunopositive cells was also analyzed in the penumbra because astrocytes could be activated mainly in this area after an ischemic challenge [21,22]. RAGE was upregulated on all determined cell types (Figure 1c,d). Among cell types determined, it was highly expressed on Iba1-positive cells compared to that on cells expressing other brain cells markers (Figure 1c,d). These results indicate that RAGE upregulation could occur mainly in activated microglia/macrophages of injured brains after an ischemic stroke.

### 3.2. Suppressing LPA_5_ Activity Attenuates RAGE Upregulation in Injured Brains after tMCAO Challenge

**Figure 2:**
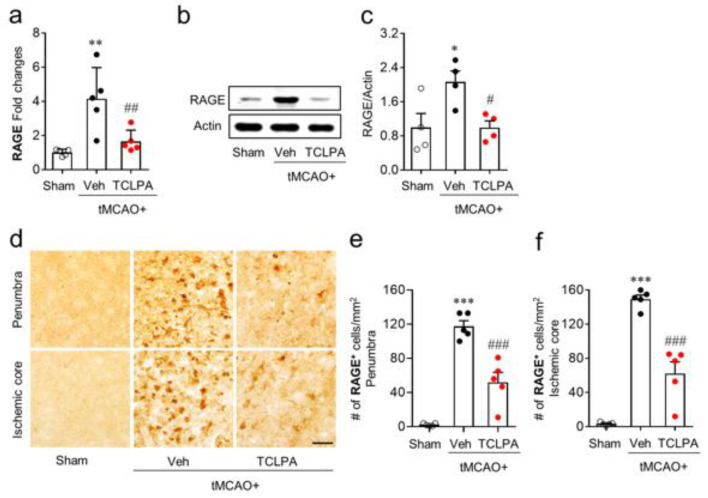
Lysophosphatidic acid receptor 5 (LPA_5_) antagonist attenuates RAGE upregulation in injured brains after tMCAO challenge. Mice were challenged with tMCAO. TCLPA5 (TCLPA: 10 mg/kg, i.p.) was administered immediately after reperfusion. (**a**) Expression levels of RAGE mRNA at 3 days after tMCAO challenge were determined by qRT-PCR analysis. n = 5 mice per group. (**b**,**c**) Expression levels of RAGE protein at 3 days after tMCAO challenge were determined by Western blot analysis. Representative blot of RAGE expression (**b**) and quantification (**c**) are shown. n = 4 mice per group. (**d**–**f**) Expression levels of RAGE protein in the penumbra and ischemic core regions at 3 days after tMCAO challenge were determined by immunohistochemical analysis. Representative images of RAGE-immunopositive cells in each region (**d**) and quantification of their numbers ((**e**), the penumbra region; (**f)**, the ischemic core region) are shown. Scale bar, 50 µm. n = 5 mice per group. * *p* < 0.05, ** *p* < 0.01, and *** *p* < 0.001 versus sham; ^#^ *p* < 0.05, ^##^ *p*< 0.01, and ^###^ *p* < 0.001 versus vehicle-administered tMCAO group.

Given data that RAGE could be upregulated in injured brains after tMCAO challenge (Figure 1), we determined whether LPA_5_ could be associated with such RAGE upregulation. To address this, we employed TCLPA5, an LPA_5_ antagonist, which could attenuate brain injuries after tMCAO challenge [9,10]. We chose 3 days after tMCAO challenge as the time point for analysis because RAGE upregulation was clearly observed at this time point (Figure 1a,b). We first determined expression levels of RAGE mRNA by qRT-PCR analysis. RAGE mRNA expression levels were significantly increased in the vehicle-administered group compared to those in the sham group (Figure 2a). TCLPA5 administration significantly attenuated such increase in injured brains (Figure 2a). Next, we determined expression levels of RAGE protein at 3 days after tMCAO challenge by Western blot analysis. The protein expression levels of RAGE were significantly upregulated in injured brains (Figure 2b,c). However, TCLPA5 administration significantly attenuated such RAGE upregulation (Figure 2b,c). Attenuated RAGE upregulation by TCLPA5 administration was reaffirmed by immunohistochemical analysis. The number of RAGE-positive cells was significantly increased in the penumbra and ischemic core regions of injured brains at 3 days after tMCAO challenge (Figure 2d–f). However, TCLPA5 administration significantly reduced the number of RAGE-positive cells in both regions (Figure 2d–f). These results indicate that LPA_5_ could regulate RAGE upregulation in injured brains after an ischemic stroke.

**Figure 3:**
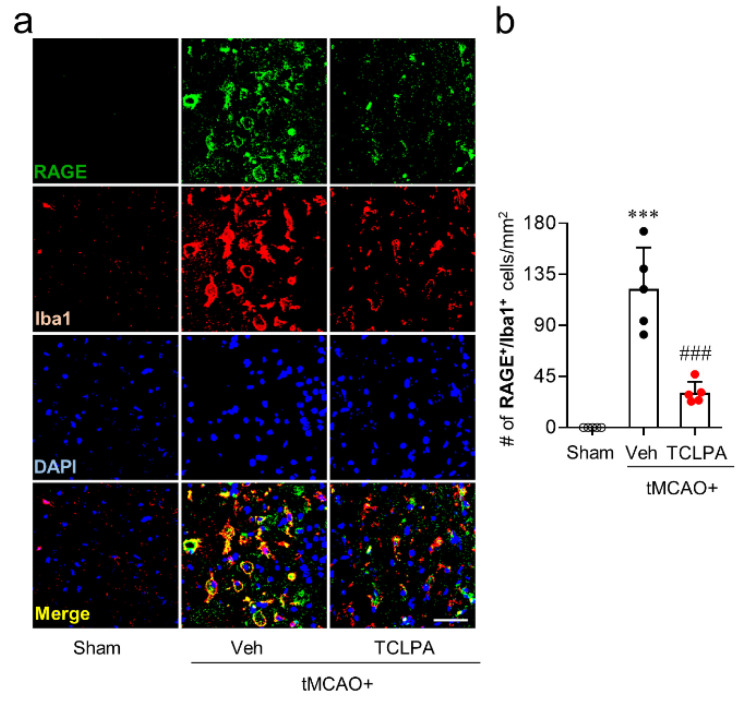
LPA_5_ antagonist attenuates RAGE upregulation on activated microglia/macrophages in injured brains after tMCAO challenge. Mice were challenged with tMCAO. TCLPA5 (TCLPA: 10 mg/kg, i.p.) was administered immediately after reperfusion. RAGE expression on activated microglia/macrophages in the ischemic core regions at 3 days after tMCAO challenge was determined by RAGE/Iba1 double immunofluorescence analysis. Representative images of RAGE/Iba1-double immunopositive cells (**a**) and quantification of their numbers (**b**) are shown. Scale bar, 50 µm. n = 5 mice per group. *** *p* < 0.001 versus sham; ^###^ *p* < 0.001 versus vehicle-administered tMCAO group.

In the current study, we demonstrated that RAGE upregulation could occur mainly in activated microglia/macrophages of injured brains after tMCAO challenge (Figure 1c,d). Therefore, we next determined whether suppressing LPA_5_ activity could attenuate RAGE upregulation on activated microglia/macrophages by RAGE/Iba1 double immunofluorescence analysis. The number of RAGE/Iba1-double immunopositive cells was significantly increased in the ischemic core regions at 3 days after tMCAO challenge (Figure 3a,b). However, TCLPA5 administration significantly reduced the number of RAGE/Iba1-double immunopositive cells (Figure 3a,b). These results indicate that LPA_5_ could upregulate expression of RAGE on activated microglia/macrophages in injured brains after an ischemic stroke.

### 3.3. Suppressing LPA_5_ Activity Attenuates BBB Disruption in Injured Brains after tMCAO Challenge

**Figure 4:**
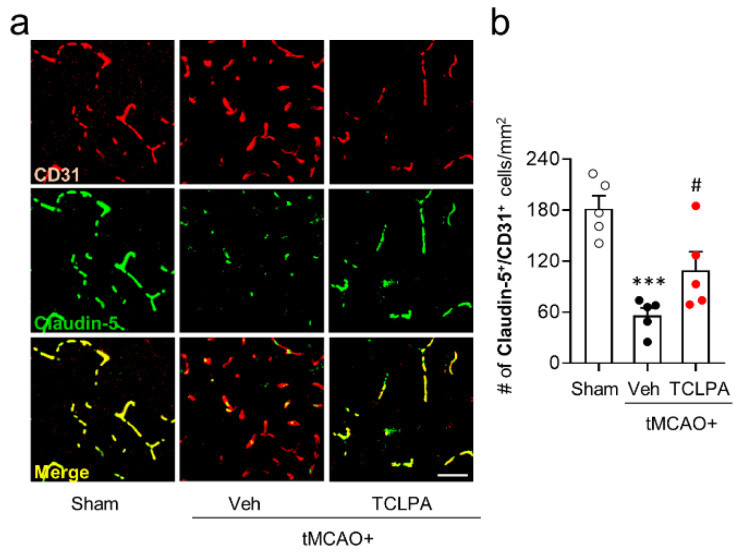
LPA_5_ antagonist attenuates blood–brain barrier (BBB) disruption in injured brains after tMCAO challenge. Mice were challenged with tMCAO. TCLPA5 (TCLPA: 10 mg/kg, i.p.) was administered to mice immediately after reperfusion. (**a**,**b**) BBB disruption at 3 days after tMCAO challenge was determined by claudin-5/Iba1 double immunofluorescence analysis. Representative images of claudin-5/CD31-double immunopositive cells in cortical regions of post-ischemic brains (**a**) and quantification of their numbers (**b**) are shown. Scale bar, 50 µm. n = 5 mice per group. *** *p* < 0.001 versus sham; ^#^ *p* < 0.05 versus vehicle-administered tMCAO group.

RAGE can contribute to BBB disruption in CNS diseases including brain ischemic stroke [15,16,23]. Therefore, we addressed roles of LPA_5_ in BBB disruption after ischemic stroke. For this, we determined whether suppressing LPA_5_ activity by TCLPA5 administration could attenuate BBB disruption in injured brains at 3 days after tMCAO challenge through claudin-5/CD31 double immunofluorescence staining. The number of claudin-5/CD31-double immunopositive cells in injured brains after tMCAO challenge was markedly decreased compared to that in the sham group (Figure 4a,b), whereas TCLPA5 administration significantly increased it (Figure 4a,b). These data indicate that LPA_5_ could contribute to BBB disruption in injured brains after an ischemic stroke.

### 3.4. LPA_5_ Mediates Activation of ERK1/2 and NF-κB in Injured Brains after tMCAO Challenge

**Figure 5:**
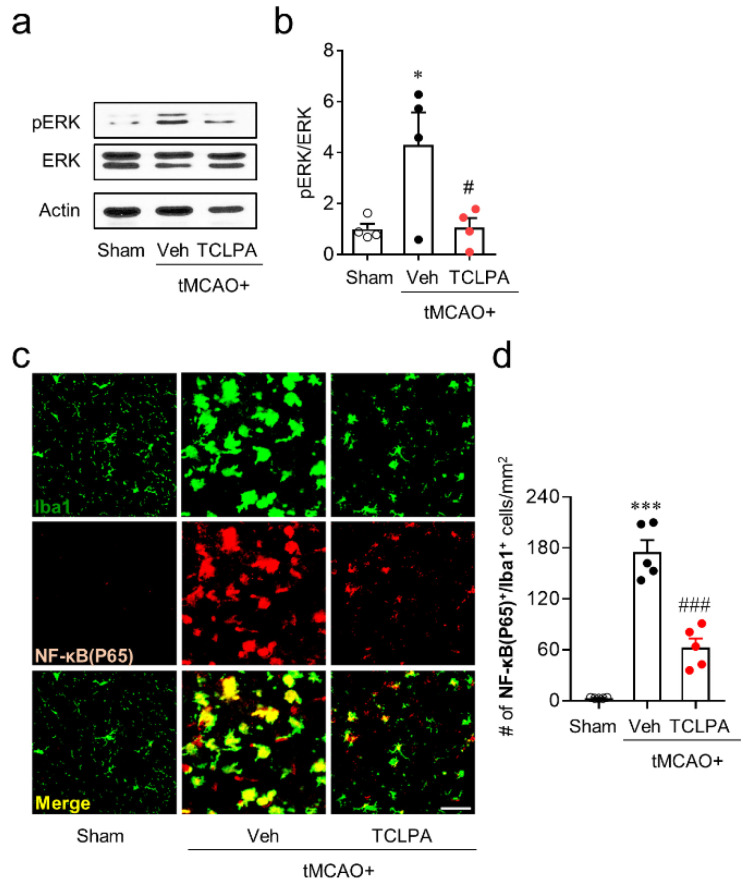
LPA_5_ antagonist attenuates activation of ERK1/2 and NF-κB in injured brains after tMCAO challenge. Mice were challenged with tMCAO. TCLPA5 (TCLPA: 10 mg/kg, i.p.) was administered to mice immediately after reperfusion. (**a**,**b**) ERK1/2 phosphorylation at 3 days after tMCAO challenge was determined by Western blot analysis. Representative blot of RAGE expression (**a**) and quantification (**b**) are shown. n = 4 mice per group. (**c**,**d**) NF-κB expression in activated microglia/macrophages at 3 days after tMCAO challenge was determined by Iba1/NF-κB double immunofluorescence analysis. Representative images of Iba1/NF-κB-double immunopositive cells in ischemic core regions (**c**) and quantification of their numbers (**d**) are shown. Scale bar, 50 µm. n = 5 mice per group. * *p* < 0.05 and *** *p* < 0.001 versus sham; ^#^ *p* < 0.05 and ^###^ *p* < 0.01 versus vehicle-administered tMCAO group.

ERK1/2 and NF-κB are known to be involved in RAGE-dependent BBB disruption [16,19]. Therefore, the role of LPA_5_ in activation of these signaling molecules in injured brains after ischemic challenge was addressed. First, we determined whether suppressing LPA_5_ activity could attenuate ERK1/2 activation in injured brains at 3 days after tMCAO challenge by Western blot analysis. ERK1/2 phosphorylation was significantly increased in injured brains after tMCAO challenge compared to that in the sham group (Figure 5a,b). When LPA_5_ activity was suppressed by TCLPA5 administration, such ERK1/2 phosphorylation was significantly attenuated (Figure 5a,b). Activated microglia/macrophages are main loci for NF-κB activation in post-ischemic brains [8,24]. The current study clearly showed RAGE upregulation on activated microglia/macrophages after tMCAO challenge (Figure 1c,d). Thus, we next determined whether suppressing LPA_5_ activity could attenuate NF-κB activation in activated microglia/macrophages of injured brains by NF-κB/Iba1 double immunofluorescence staining. The number of NF-κB/Iba1-double immunopositive cells was significantly increased in injured brains at 3 days after tMCAO challenge compared to that in the sham group (Figure 5c,d). However, suppressing LPA_5_ activity by TCLPA5 administration significantly reduced their number in injured brains (Figure 5c,d). These results indicate that LPA_5_ could regulate activation of ERK1/2 and NF-κB in injured brains after an ischemic stroke.

## 4. Discussion

LPA_5_ can contribute to acute brain injuries along with neuroinflammatory responses in injured brains after ischemic stroke, including activation of microglia/macrophages and production of proinflammatory cytokines [9]. Suppressing LPA_5_ activity with its antagonist, TCLPA5, can attenuate such acute brain injuries and neuroinflammatory responses in mice after ischemic challenge [9]. In addition, it can attenuate chronic brain injuries in mice after ischemic challenge by enhancing neurogenesis and angiogenesis [10]. These two independent studies clearly suggest that LPA_5_ can play pathogenic roles in brain injuries during both acute and chronic phases after ischemic stroke. Results of the current study suggest that RAGE is a pathogenic factor for LPA_5_-mediated brain injuries after ischemic stroke. RAGE was upregulated during the acute phase after ischemic challenge (at 1 day and 3 days after tMCAO challenge) in the penumbra and ischemic core regions, particularly on activated microglia/macrophages, whereas suppressing LPA_5_ activity with TCLPA5 attenuated such a RAGE upregulation. Suppressing LPA_5_ activity with TCLPA5 also attenuated RAGE-relevant BBB disruption in injured brains after tMCAO challenge. As underlying signaling pathways, LPA_5_ could be associated with activation of ERK1/2 and NF-κB, both of which are effector pathways of RAGE [25,26]. They might be involved in BBB disruption [27]. All of these data clearly suggest that LPA_5_ could regulate the pathogenesis of brain ischemic stroke via RAGE, at least in part.

It is well-known that RAGE plays pathogenic roles in various diseases [11,23], including brain ischemic stroke [14,15,16]. Suppressing RAGE activity by either a pharmacological antagonist or genetic deletion can attenuate ischemic brain injuries including brain infarction, neurological functional deficit, and neuronal survival in different rodent models such as permanent MCAO (pMCAO)-challenged mice [14], intracerebral hemorrhage (ICH)-challenged rats [16], and bilateral common carotid artery occlusion (BCCAO)-challenged mice [15]. Such a pathogenic role of RAGE is supported by its upregulation at both mRNA and protein levels in injured brains of ischemic stroke-challenged rodents [15,16,20] and human stroke patients [20]. In addition, immunohistochemical analyses have suggested regional and cell type-specific RAGE upregulation after ischemic challenge [15,16,20]. The penumbra region has been demonstrated to be the area that RAGE is upregulated after an ischemic challenge in pMCAO-challenged mice [20]. In view of cell types, endothelial cells, activated microglia/macrophages, and neurons have been suggested as cell types where RAGE can be upregulated after ICH or BCCAO challenge [15,16]. In the current study using tMCAO-challenged mice, RAGE was upregulated in post-ischemic brains at 1 day and 3 days after tMCAO challenge, with higher expression levels at 3 days after tMCAO challenge than at 1 day after such challenge. Interestingly, RAGE upregulation was observed in the penumbra and ischemic core regions, but not in periischemic regions, with a stronger immunoreactivity for RAGE in the ischemic core region than in the penumbra region. Moreover, the current study revealed that RAGE upregulation occurred mainly in activated microglia/macrophages of injured brains after tMCAO challenge. This could be supported by previous studies showing that RAGE is upregulated on activated microglia/macrophages in injured brains after ICH challenge [16] or on cultured microglia subjected to oxygen and glucose deprivation as an in vitro ischemic challenge [14]. In addition to activated microglia/macrophages, the current study demonstrated that RAGE could be upregulated on neurons and endothelial cells of injured brains after tMCAO challenge as demonstrated in previous studies using other stroke models [15,16]. All these independent studies including ours strongly indicate that RAGE can be upregulated in injured brains after ischemic challenge, leading to ischemic brain injuries. It is of note that LPA_5_ can lead to RAGE upregulation in post-ischemic brains. Suppressing LPA_5_ activity with TCLPA5 administration attenuated RAGE upregulation at mRNA and protein levels in injured brains after tMCAO challenge. Moreover, it attenuated RAGE upregulation in both the penumbra and ischemic core regions, particularly on activated microglia/macrophages, after tMCAO challenge. Considering that our previous studies have shown that LPA_5_ could contribute to tMCAO-induced brain damages [9], the current study indicates that RAGE can be involved in LPA_5_-mediated ischemic brain injuries as an underlying pathogenic mediator. Interestingly, LPA_5_ can be upregulated in the ischemic core regions, particularly on activated microglia/macrophages, but not on neurons [9]. Therefore, it might be possible that LPA_5_ regulate RAGE expression in activated microglia/macrophages after ischemic challenge.

RAGE is known to regulate inflammatory responses through activation of ERK1/2 and NF-κB [25,26], both of which are associated with BBB disruption [27], indicating that RAGE can contribute to BBB disruption through activation of these signaling molecules. In fact, suppressing RAGE activity with FPS-ZM1, a RAGE-specific antagonist, can significantly attenuate BBB dysfunction in radiation-induced endothelial barrier injury through inactivation of ERK1/2 and NF-κB [19]. It can also attenuate BBB disruption in injured brains after ICH challenge, along with downregulation of NF-κB and proinflammatory mediators such as interleukins, inducible nitric oxide synthase, cyclooxygenase-2, and matrix metalloproteinase-9 [16]. Similarly, the current study demonstrated that suppressing LPA_5_ activity with TCLPA5 administration could attenuate BBB disruption, ERK1/2 activation, and NF-κB activation in injured brains after tMCAO challenge, indicating that suppressing RAGE expression by inhibiting LPA_5_ activity might lead to such attenuated events. The current study also demonstrated that NF-κB activation and RAGE upregulation occurred in activated microglia/macrophages after tMCAO challenge whereas TCLPA5 administration attenuated both events. In fact, activated microglia/macrophages can contribute to BBB disruption in many brain diseases, including brain ischemic stroke [28,29]. Moreover, LPA_5_ can contribute to the activation of microglia/macrophages and subsequent neuroinflammatory responses, such as upregulation of proinflammatory cytokines (TNF-α, IL-1β, and IL-6) in post-ischemic brains [9]. These proinflammatory cytokines have been considered as contributing factors for BBB disruption after ischemic challenge [30,31]. Therefore, RAGE might play important roles in LPA_5_-mediated BBB disruption and neuroinflammatory responses in post-ischemic brains possibly by regulating the activation of ERK1/2 and NF-κB.

## 5. Conclusions

The current study demonstrates that RAGE can be involved in LPA_5_-mediated brain injury following ischemic stroke, along with experimental evidence for roles of LPA_5_ in RAGE-relevant BBB disruption and signaling pathways, such as ERK1/2 and NF-κB in post-ischemic brains. LPA_5_ has been suggested as a pathogenic mediator for other disease types, such as neuropathic pain [32,33], psoriasis [34], demyelination [35], and itching [36]. Findings of the current study on a possible involvement of RAGE in LPA_5_-mediated pathogenesis in ischemic stroke might add clues for understanding how LPA_5_ can play its pathogenic roles in such diseases. In addition, targeting LPA_5_ might be a possible strategy to treat various diseases involving RAGE as a pathogenic factor.

## Abbreviations

Aβ	Amyloid beta
AGEs	Advanced glycation end products
BBB	Blood–brain barrier
BCCAO	Bilateral common carotid artery occlusion
CCA	Common carotid artery
DAB	3,3′-Diaminobenzidine
DAPI	4′,6-Diamidino-2-phenylindole
HMGB1	High mobility group box 1 non-histone DNA-binding protein
ICH	Intracerebral hemorrhage
LPA	Lysophosphatidic acid
LPA_1_	Lysophosphatidic acid receptor 1
LPA_5_	Lysophosphatidic acid receptor 5
PFA	Paraformaldehyde
pMCAO	Permanent middle cerebral artery occlusion
qRT-PCR	Quantitative real-time PCR
RAGE	Receptor for advanced glycation end products
tMCAO	Transient middle cerebral artery occlusion
